# *Xist* Exon 7 Contributes to the Stable Localization of Xist RNA on the Inactive X-Chromosome

**DOI:** 10.1371/journal.pgen.1005430

**Published:** 2015-08-05

**Authors:** Norishige Yamada, Yuko Hasegawa, Minghui Yue, Tomofumi Hamada, Shinichi Nakagawa, Yuya Ogawa

**Affiliations:** 1 Division of Reproductive Sciences, Cincinnati Children’s Hospital Medical Center, Cincinnati, Ohio, United States of America; 2 Department of Pediatrics, University of Cincinnati College of Medicine, Cincinnati, Ohio, United States of America; 3 RNA Biology Laboratory, RIKEN Advanced Research Institute, Wako, Saitama, Japan; 4 Department of Oral Surgery, Kagoshima University Hospital, Kagoshima, Japan; Massachusetts General Hospital, Howard Hughes Medical Institute, UNITED STATES

## Abstract

To equalize X-linked gene dosage between the sexes in mammalian females, Xist RNA inactivates one of the two X-chromosomes. Here, we report the crucial function of *Xist* exon 7 in X-inactivation. *Xist* exon 7 is the second-largest exon with a well-conserved repeat E in eutherian mammals, but its role is often overlooked in X-inactivation. Although female ES cells with a targeted truncation of the *Xist* exon 7 showed no significant differences in their *Xist* expression levels and RNA stability from control cells expressing wild-type *Xist*, compromised localization of Xist RNA and incomplete silencing of X-linked genes on the inactive X-chromosome (Xi) were observed in the exon 7-truncated mutant cells. Furthermore, the interaction between the mutant Xist RNA and hnRNP U required for localization of Xist RNA to the Xi was impaired in the *Xist* exon 7 truncation mutant cells. Our results suggest that exon 7 of Xist RNA plays an important role for stable Xist RNA localization and silencing of the X-linked genes on the Xi, possibly acting through an interaction with hnRNP U.

## Author Summary

To balance gene expression from X-chromosomes between males and females, one of the two X-chromosomes is inactivated in female mammals. X-chromosome inactivation is a chromosome-wide epigenetic gene silencing mechanism regulated by long non-coding Xist RNA. Mouse Xist RNA is commonly organized into 7 exons, with the extensively studied and known important domains of *Xist* residing within exon 1. However, the function of exon 7 of Xist RNA, which is the second longest exon, remains poorly understood. Our objective was to clarify the role of this exon in X-inactivation through the use of *Xist* truncation mutant female ES cells. Here, we provide evidence that *Xist* exon 7 is required for the stable localization of Xist RNA and X-linked gene silencing on the inactive X-chromosome.

## Introduction

In eukaryotes, an overwhelming majority of genomes are transcribed as non-protein-coding RNAs (ncRNAs) [1,2]. One of the major classes of ncRNAs is long ncRNAs (lncRNAs), which vary in length from a few hundred bases to tens of kilobases. A number of lncRNAs play an important role in transcriptional regulation through their interaction with chromatin-modifying enzymes, which direct them to specific target genes [3,4]. LncRNAs are also known to be involved in various biological processes such as the regulation of the cell cycle [5], cellular differentiation and development [6], the regulation of metabolism [7] and disease pathogenesis [8,9].

X inactive-specific transcript (Xist) RNA is one such lncRNA, which regulates chromatin organization and transcriptional gene silencing in one of the two X-chromosomes to equalize the X-linked gene dosage between males and females [10]. In the epiblast lineage, either the paternal or maternal X is randomly inactivated; this is referred to as random X-inactivation. In random X-inactivation, several non-coding genes in the X-inactivation center (*Xic*) are known to interact to initiate X-inactivation in one of the two X-chromosomes in females [11,12]. Xist RNA is exclusively expressed from the *Xic* of the future inactive X-chromosome (Xi) at the onset of X-inactivation and has a pivotal role in initiating X-inactivation *in cis* [13]. Highly expressed Xist RNA coats the entire length of Xi [14] and recruits silencing factors such as the polycomb repressive complex 2 (PRC2) of lysine methyltransferase for H3K27me3 to the Xi [15,16]. Xist RNA induces gene silencing on the Xi by a cascade of epigenetic modifications that are maintained through multiple rounds of cell division [17]. There is evidence that the overall X-inactivation can be maintained in the absence of *XIST*/*Xist* [18,19]; however, recent evidence has shown that *Xist* deletion from the Xi induces the partial de-repression of the X-linked genes [17,20]. Indeed, a recent paper has shown that the depletion of *Xist* in murine hematopoietic stem cells after the establishment of X-inactivation leads to a genome-wide aberration in gene expression, especially in the expression of X-linked genes, and an induction of highly aggressive myeloproliferative neoplasm and myelodysplastic syndrome in a female-specific manner [21]. This finding suggests a critical role of *Xist* in the maintenance phase of X-inactivation to prevent cancer transformation and progression. Therefore, proper regulation of *Xist* is critical in both the initiation and maintenance phases for cell survival, cellular differentiation and development, and the prevention of cancer pathogenesis in mammalian species.

Xist RNA has multiple functional domains and directly or indirectly interacts with various proteins such as transcription factors, chromatin modifying enzymes and scaffold proteins [22]. Comprehensive functional analysis using a series of deletions of Xist RNA based on the inducible *Xist* transgene has identified the functional domain of Xist RNA for gene silencing, and broad redundant region for Xist RNA localization on the Xi and formation of macrochromatin bodies (MCB) associated with histone variant macroH2A1 [23]. This approach successfully demonstrated that repeat A of the 5´ region of Xist RNA is crucial for X-linked gene silencing and that the redundant region contributes to stable Xist RNA localization on the Xi. Although PRC2 binds promiscuously to a variety of RNA molecules, PRC2 exhibits preferential binding to repeat A of the Xist RNA in vivo and in vitro [24–31]. Several reports have indicated that *Xist*-specific repeat motifs contained within exon 1 and conserved in various eutherian mammalian species are involved in the localization of Xist RNA on the Xi [32–34]. Transcriptional factor YY1 interacts with both Xist RNA and the *Xist* gene body through repeat C of Xist RNA and YY1 binding sites near repeat F in *Xist*, respectively; it is believed to anchor the Xist RNA to the *Xist* gene on the Xi as a nucleation center for Xist RNA spreading [34]. Moreover, the interaction between Xist RNA and the Xi can be blocked by targeting *Xist* repeat C with peptide nucleic acids (PNAs) or locked nucleic acids (LNAs). This fact contrasts with findings that a lack of repeat C does not affect the localization of Xist RNA on the Xi of the transgene assay [23,32,33].

Heterogeneous nuclear ribonucleoprotein U (hnRNP U; also known as SAF-A or SP120) has an essential role for Xist RNA localization on the Xi [35]. Interestingly, hnRNP U accumulation on the Xi is also dependent on the Xist RNA, indicating an interdependent relationship between Xist RNA and hnRNP U for their specific localization on the Xi [35,36]. hnRNP U was originally identified as a nuclear matrix- or scaffold-attachment region (MAR or SAR)-associated protein [37–39]. It is known to have a wide variety of functions such as gene expression, telomere regulation and nuclear organization [40–43]. Because hnRNP U has a unique molecular structure with a SAF-Box MAR binding domain and an arginine-glycine-glycine (RGG) RNA-binding domain at its N-terminal and C-terminal, respectively [44,45], hnRNP U has been proposed to bridge Xist RNA and the Xi [35].

While most Xist RNA functional domains for RNA localization and gene silencing are mapped within exon 1 of Xist RNA, the function of exon 7 in Xist RNA is unknown in spite of the presence of a well-conserved repeat E among the eutherian mammals [46]. To elucidate the role of the *Xist* exon 7 in X-inactivation, we generated *Xist* exon 7-truncated mutant ES cells by inserting tandem polyadenylation (tpA) signals. In this paper, we report our novel findings regarding the interplay between the *Xist* exon 7 and hnRNP U for stable localization of Xist RNA on the Xi and X-linked gene silencing.

## Results

### A truncation mutation of Xist RNA at the end of exon 6 does not affect the expression or stability of Xist RNA during X-inactivation

**Fig 1:**
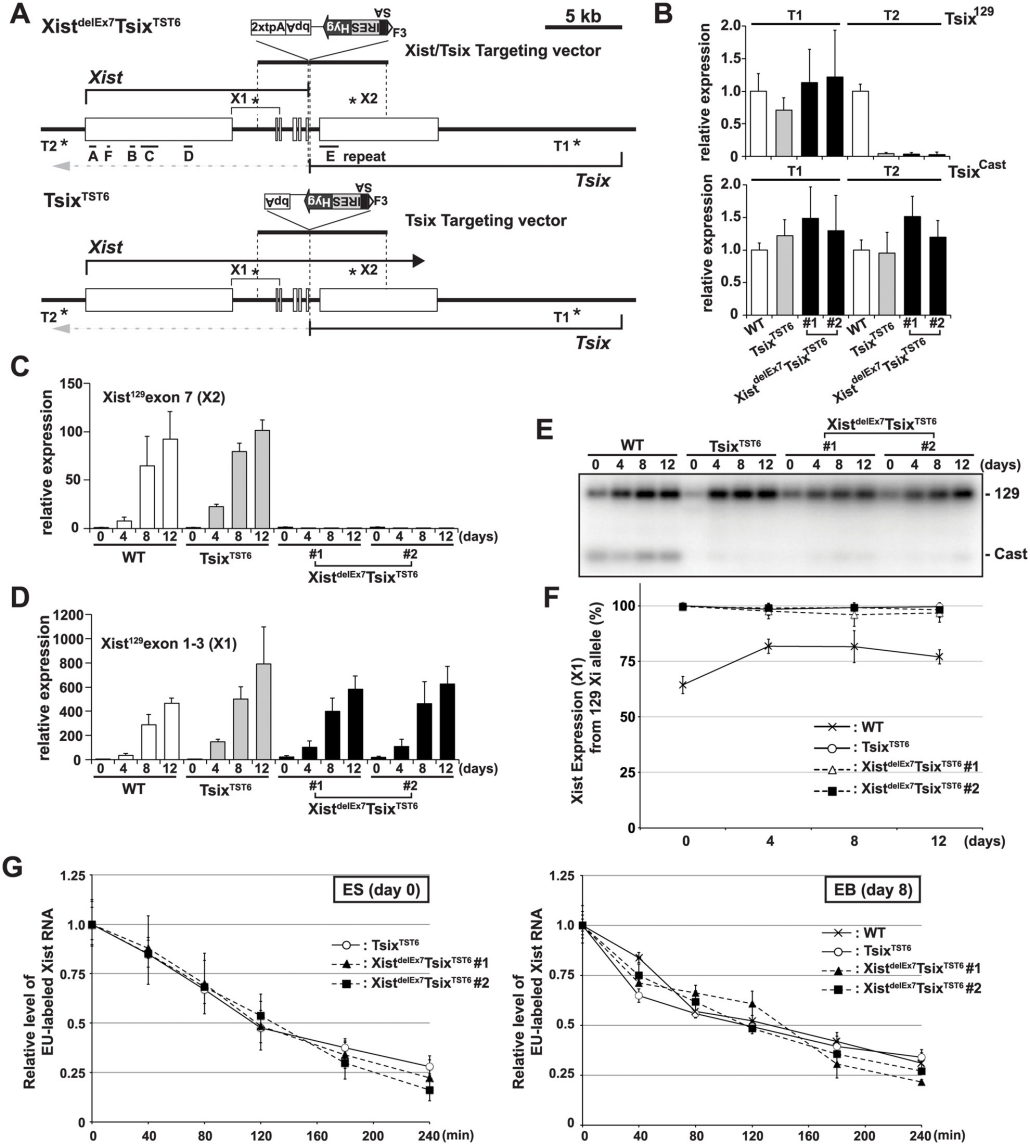
The creation of the *Xist*/*Tsix* double truncation Xist^delE7^Tsix^TST6^ and *Tsix* truncation Tsix^TST6^ mutant female ES cells. (A) A map of the *Xist*/*Tsix* locus. The positions of the primer pairs used for RT-PCR are indicated with asterisks. SA, splice acceptor; IRES, internal ribosome entry site; Hyg, hygromycin resistance gene; bpA, beta-actin polyadenylation signal; tpA, tandem polyadenylation signal. (B) 129 and Cast allele-specific RT-qPCR analysis for Tsix RNA at positions T1 and T2, as shown Fig 1A, in Tsix^TST6^ and two Xist^delE7^Tsix^TST6^ undifferentiated ES cells (#1 and #2). Each value was normalized to that of the wild-type cells (WT) (set to 1), and Gapdh was used as an internal control. Values are given as the mean ± standard deviation (SD) of three independent experiments. (C and D) 129 allele-specific RT-qPCR analysis of the *Xist* expression at exons 7 and exons 1–3, respectively, was conducted. The expression values were normalized to those of WT at day 0 (set to 1) and Gapdh. The mean ± SD from three independent experiments is shown. (E) Representative allele-specific RT-PCRs for *Xist*. (F) Quantitative analysis of allele-specific RT-PCRs from three independent experiments including Fig 1E (mean ± SD). (G) Half-life assay for Xist RNA in undifferentiated ES cells and differentiated EBs on day8 upon differentiation. The mean ± SD values from two independent experiments are shown.

To investigate the role of exon 7 in mouse Xist RNA, we inserted two tandem polyadenylation signals (2xtpA) into the end of exon 6 of *Xist* coupled with splice acceptor (SA)-internal ribosomal entry site (Ires)-hygromycin (Hyg)-pA for *Tsix* truncation to induce non-random X-inactivation of the mutant X. This resulted in the simultaneous truncation of both *Xist* and *Tsix* (Xist^delEx7^Tsix^TST6^) (Fig 1A and S1 Fig). We used the 16.7 mouse female ES cell line carrying one *Mus musculus* 129SvJ (129) X-chromosome and one *Mus castaneous* (Cast) X, which enabled us to perform allele-specific analysis based on single nucleotide polymorphisms (SNPs) between these mouse strains [47,48]. As a control, we also established a *Tsix* truncation mutant by inserting SA-Ires-Hyg-pA at *Xist* intron 6, resulting in Tsix^TST6^ female ES cells (Fig 1A and S1 Fig). Allele-specific quantitative RT-PCR (RT-qPCR) analysis of the *Tsix* expression revealed that the 129 X-chromosome was targeted by the truncation mutation (Fig 1B). While the *Tsix* expression upstream of the truncation site could be detected by using both the 129 and Cast allele-specific primer sets (T1) in control Tsix^TST6^ and two Xist^delEx7^/Tsix^TST6^ mutant cells, the Tsix transcript was efficiently truncated downstream (T2, *Tsix* ^*129*^ exon 4) of the pA insertion site. These mutant ES cells allow us to address the effect of the *Xist* mutation on X-inactivation because the *Tsix* mutation caused a non-random inactivation of the mutant 129 X-chromosome [47].

First, to determine if the 2xtpA insertion was sufficient for the truncation of the Xist RNA, we performed 129 allele-specific RT-qPCR for the *Xist* exon 7 (Fig 1C). In two independently isolated Xist^delEx7^Tsix^TST6^ mutant clones, the *Xist* exon 7 on the 129 Xi was found to not be expressed upon *ex vivo* differentiation. This suggests that the insertion of the 2xtpA cassettes efficiently truncates the Xist transcripts during X-inactivation. Next, to investigate the Xist expression in the Xist^delEx7^Tsix^TST6^ mutant cells upon differentiation, the 129 allele-specific RT-qPCR was carried out using a primer set designed to amplify *Xist* exon 1 through exon 3 (Fig 1D). This RT-qPCR showed that *Xist* expression was upregulated in both Tsix^TST6^ and Xist^delEx7^Tsix^TST6^ mutant cells upon differentiation, comparable to the *Xist* expression level in wildtype female cells. We also examined the ratio of Xist RNA expressed from mutant 129 Xi to that from Cast wild-type active X-chromosome (Xa) by allele-specific RT-PCR analysis using a polymorphic restriction enzyme digestion with wild-type, control Tsix^TST6^ and Xist^delEx7^Tsix^TST6^ mutant cells (Fig 1E and 1F). As expected, due to the *Tsix* mutation, the *Xist* was nearly exclusively expressed in the 129 X-chromosomes of both the Tsix^TST6^ and Xist^delEx7^Tsix^TST6^ mutant cells in contrast to the wild-type cells (70–80% of the 129 X was chosen as the Xi) [48]. Furthermore, because the *Xist* truncation mutant deletes approximately 40% of full length Xist RNA (7.5 kb out of 17.8 kb), we investigated whether an *Xist* truncation mutation lacking exon 7 affects the stability of the RNA by measuring the half-lives of 5-ethynyl uridine (EU) pulse-labeled Xist RNAs (Fig 1G). The half-life assay identified no significant differences in the Xist RNA stability between the control and two mutant clones. The half-life of the full-length wild-type Xist RNA in Tsix^TST6^ ES cells and the exon 7-truncated mutant Xist RNA in the two Xist^delEx7^Tsix^TST6^ ES cells was approximately 2 hours. This half-life shorter than that observed in previous reports, which found a half-life of ~3.5 hours in the Xist RNA half-life assay of the *Tsix* knockout mutant female ES cells using RNA polymerase II inhibitor. This differences can likely be explained by the different approaches we used [49]. Similar to the half-life of the wild-type and exon 7-truncated mutant Xist RNA in ES cells, the half-life of the wild-type and mutant Xist RNA in EB upon differentiation on day 8 was approximately 2~2.5 hours (Fig 1G). These results suggest that the truncation of the *Xist* exon 7 does not alter the *Xist* expression or the stability of the Xist RNA during X-inactivation. Because a shorter isoform (S-isoform) of the Xist RNA lacks a large region of exon 7 in a long isoform (L-isoform) except for a 1.1 kb repeat E and 0.5 kb region of its 3´ end by alternative splicing, the S-isoform Xist RNA is similar to the mutant Xist^delEx7^ RNA lacking the entire exon 7. We examined whether the L- and S-isoforms Xist RNA have a different stability. The L-isoforms of the Xist RNA exhibited a slightly longer half-life in wildtype 16.7 and cells TsixTST6 mutant (S2 Fig), suggesting that alternative splicing might slightly affect the stability of Xist RNA.

Because the Xist expression is tightly linked to the differentiation status, we also performed RT-qPCR analysis of the pluripotent cell markers, Nanog and Oct3/4, to clarify whether the *Xist* truncation mutation influences the ES cell differentiation (S3A Fig). RT-qPCR showed that the Nanog and Oct3/4 expression levels in both the control Tsix^TST6^ and Xist^delEx7^Tsix^TST6^ mutant cells were similarly decreased as the embryoid body (EB) differentiation progressed. Moreover, the outgrowth of the Xist^delEx7^Tsix^TST6^ mutant cells was similar to that of the control Tsix^TST6^ mutant cells at day 8 upon differentiation, with a slightly slower growth (S3B Fig). These results suggest that EB differentiation was not significantly affected by the *Xist* exon 7 truncation mutation.

### *Xist* exon 7 is essential for the stable localization of Xist RNA on the Xi

**Fig 2:**
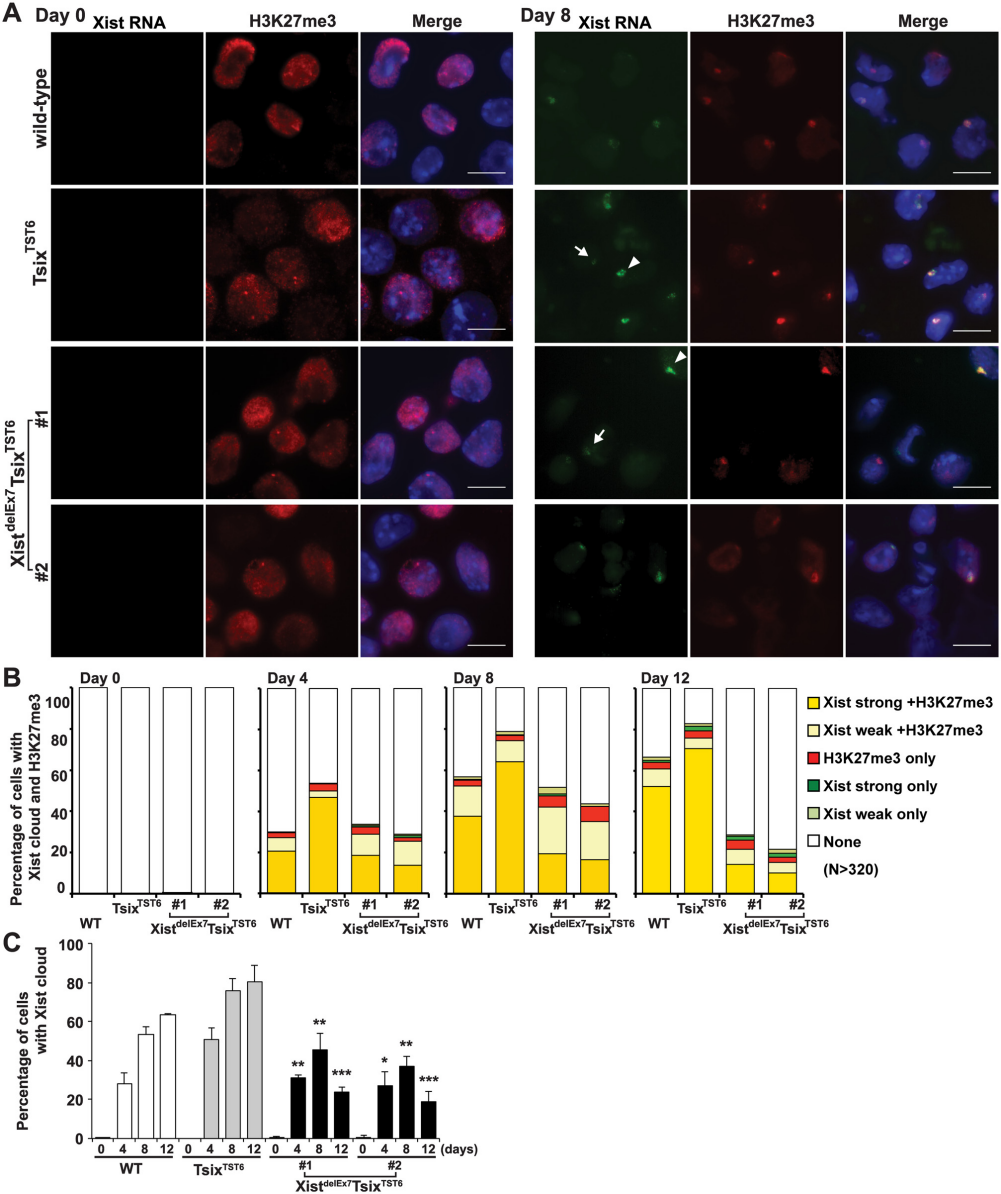
*Xist* exon 7 is required for the chromosome-wide localization of Xist RNA and H3K27me3 during X-inactivation. (A) Immuno-FISH for Xist RNA (green) and H3K27me3 (red) before (day 0) and after (day 8) differentiation. Nuclei were counterstained with DAPI. The white arrowhead and arrow indicate representative “strong” and “weak” Xist clouds classified in Fig 2B, respectively. Scale bar, 10 μm. (B) Frequency of Xist cloud- and H3K27me3-positive cells upon differentiation from three independent experiments. More than 350 nuclei in each ES cell line at each time point from three independent experiments were counted and classified based on the Xist RNA and H3K27me3 signal. Robust and faint Xist RNA FISH signals were classified as “strong” and “weak” Xist RNA, respectively. (C) The graph shows the mean ± SD of nuclei with Xist RNA clouds from three independent experiments. *P*-values were derived from an unpaired t-test between control Tsix^TST6^ and Xist^delE7^Tsix^TST6^ cells on the same day upon differentiation (*p<0.05, **p<0.01, ***p<0.001).

Having shown that neither the control Tsix^TST6^ nor the Xist^delEx7^Tsix^TST6^ mutation affected the expression of *Xist* or the stability of Xist RNA and that neither mutant exhibited severe growth defects in EB differentiation during X-inactivation, we next examined the chromosome-wide gene silencing induced by the mutant Xist RNA during X-inactivation. We performed RNA fluorescence in situ hybridization with immunofluorescence (immuno-RNA FISH) to observe the accumulation of Xist RNA and a hallmark of facultative heterochromatin marker, H3K27me3, on the Xi upon differentiation. In the Tsix^TST6^ mutant cells, the number of robust (strong) Xist RNA cloud- and H3K27me3-positive cells gradually increased upon differentiation. This is greater than that in the wild-type 16.7 ES cells at each time point, likely due to faster Xist induction by the Tsix mutation in Tsix^TST6^ mutant cells [48]. In contrast to the strong focal staining of the Xist RNA and H3K27me3 in the control Tsix^TST6^ mutant cells, the immuno-RNA FISH results in the Xist^delEx7^Tsix^TST6^ mutant cells indicated a significantly reduced number of Xist RNA cloud- and H3K27me3-positive cells, despite the comparable levels of Xist expression between the mutant and control Tsix^TST6^ cells (Figs 1D and 2). Instead of strong focal Xist RNA clouds, the Xist^delEx7^Tsix^TST6^ mutant cells often exhibited a faint (weak) Xist signal. While the Tsix^TST6^ mutant cells exhibited strong Xist clouds associated with H3K27me3 staining in approximately 65~70% of the cells on both day 8 and day 12, the co-localization of strong Xist clouds and H3K27me3 in the Xist^delEx7^Tsix^TST6^ mutant cells was less than 20% and 14% on days 8 and 12, respectively. The difference in terms of the percentages of the Xist cloud-positive nuclei between the Tsix^TST6^ and Xist^delEx7^Tsix^TST6^ mutant cells became more significant as differentiation progressed (Fig 2B and 2C). Notably, the total percentage of the Xist cloud-positive (strong and weak) cells in the Xist^delEx7^Tsix^TST6^ mutant cells on days 8 and 12 were significantly decreased from 45% to 24% (clone #1) and from 37% to 19% (clone #2), respectively. In contrast, the Tsix^TST6^ cells maintained a high percentage of Xist cloud-positive cells (over 75% Xist cloud-positive) during the transitional period from day 8 to day 12. Our results suggest that exon 7 of the Xist RNA is essential for stable Xist RNA localization, especially in the maintenance of Xist RNA localization and associated H3K27me3 modifications on the Xi.

### *Xist* exon 7 is required for the silencing of X-linked genes during X-inactivation

**Fig 3:**
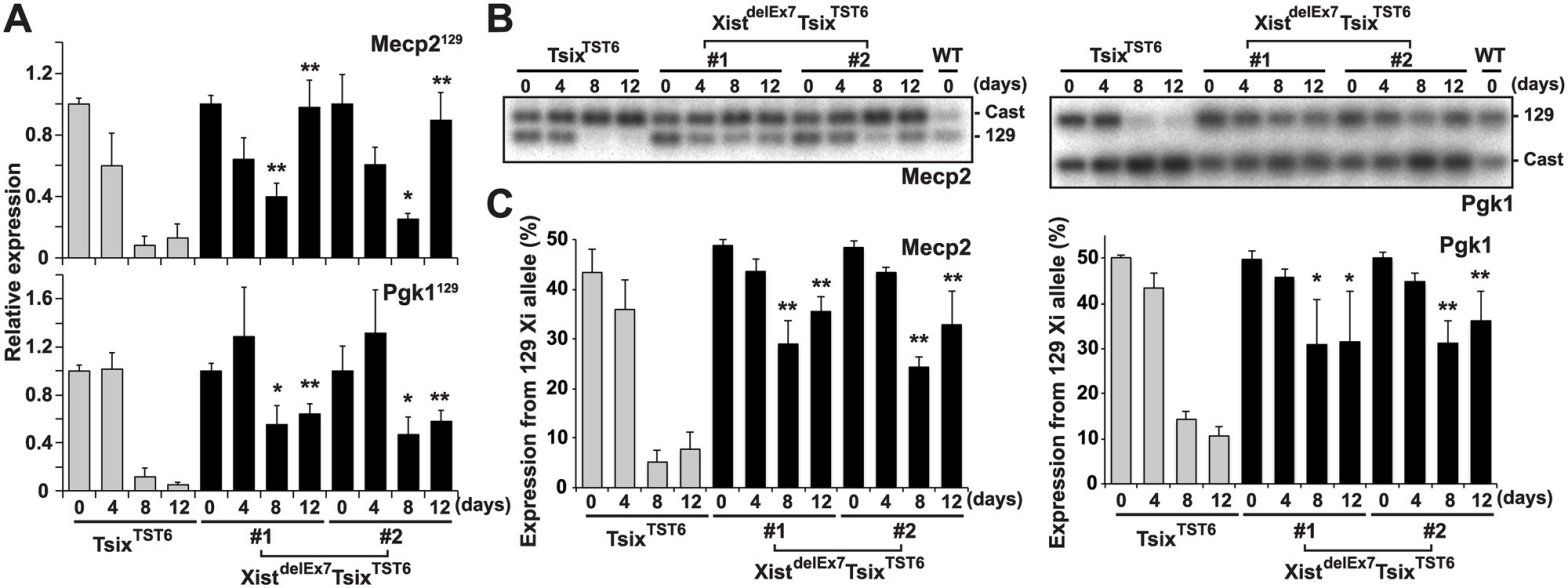
Truncation of exon 7 of Xist RNA affects the X-linked gene silencing on the Xi during EB differentiation. (A) 129 mutant allele-specific RT-qPCR analysis of X-linked *Mecp2* and *Pgk1* genes upon differentiation; each was normalized to undifferentiated cells (set to 1) and Gapdh. The mean ± SD values from three independent experiments are shown with the unpaired t test *P* values (*p<0.05, **p<0.01). (B) Representative allele-specific RT-PCR analysis for *Mecp2* and *Pgk1*. (C) Quantification of relative expression levels of *Mecp2* and *Pgk1* from the 129 allele. The mean ± SD values from the three independent experiments included in Fig 3B are shown. *P*-values were derived from an unpaired t-test (*p<0.05, **p<0.01).

Intriguingly, the Xist^delEx7^Tsix^TST6^ mutant clones exhibited impaired localization of the Xist RNA and H3K27me3 on the Xi. This observation prompted us to investigate whether the compromised Xist RNA and H3K27me3 localization on the Xi affects the expression status of X-linked genes on the Xi during EB differentiation using two different approaches (Fig 3): RT-qPCR analysis using allele-specific primers (Fig 3A) and allele-specific RT-PCR using a polymorphic restriction enzyme digestion (Fig 3B and 3C). The allele-specific RT-qPCR analysis using 129 allele-specific primer sets can discriminate the Pgk1 and Mecp2 expression on the mutant 129 X-chromosome from their expression on the Cast X-chromosome. Thus, we can only detect Pgk1 and Mecp2 expression on the mutant 129 X-chromosome. In the control Tsix^TST6^ EB cells on days 8 and 12 of differentiation, the X-linked *Mecp2* and *Pgk1* expression from the 129 Xi gradually decreased upon differentiation and remained less than 13% and 12% compared to that of the undifferentiated ES cells (day 0), respectively. In contrast to the control cells, the mutant 129 allele-specific RT-qPCR for *Mecp2* and *Pgk1* in the Xist^delEx7^Tsix^TST6^ mutant clones showed that the partial silencing of *Mecp2* (40% and 25% in clones #1 and #2, respectively) and *Pgk1* (55% and 47% in clones #1 and #2, respectively) occurred on day 8 instead of on day 0. The partial silencing of *Mecp2* and *Pgk1* in the Xist^delEx7^Tsix^TST6^ mutant cells was then followed by the reactivation of *Mecp2* (98% and 89% in clones #1 and #2, respectively) and *Pgk1* (64% and 58% in clones #1 and #2, respectively) on day 12 upon differentiation, although the initial phase of silencing in *Mecp2* and *Pgk1* on day 4 was similar between the Tsix^TST6^ and Xist^delEx7^Tsix^TST6^ mutant cells. Additionally, to confirm the compromised *Mecp2* and *Pgk1* expression from the 129 Xi relative to that of the wild-type Cast Xa in the Xist^delEx7^Tsix^TST6^ mutant cells, allele-specific RT-PCR analysis based on a polymorphic restriction enzyme digestion was performed (Fig 3B and 3C). The ratios of the X-linked gene expression from the 129 Xi were markedly reduced from differentiation day 8 in the Tsix^TST6^ cells. However, in the mutant clones, the ratios of X-linked gene expression from the 129 Xi remained high during the late stages of X-inactivation. Combined with the data in Fig 2, these results suggest that the Xist^delEx7^Tsix^TST6^ mutant Xist RNA can partially induce the silencing of X-linked genes, but cannot sustain transcriptional repression on the Xi. This is likely due to the loss of the Xist RNA clouds and the H3K27me3 on the Xi.

### hnRNP U directly interacts with exon 1 and 7 regions of Xist RNA

**Fig 4:**
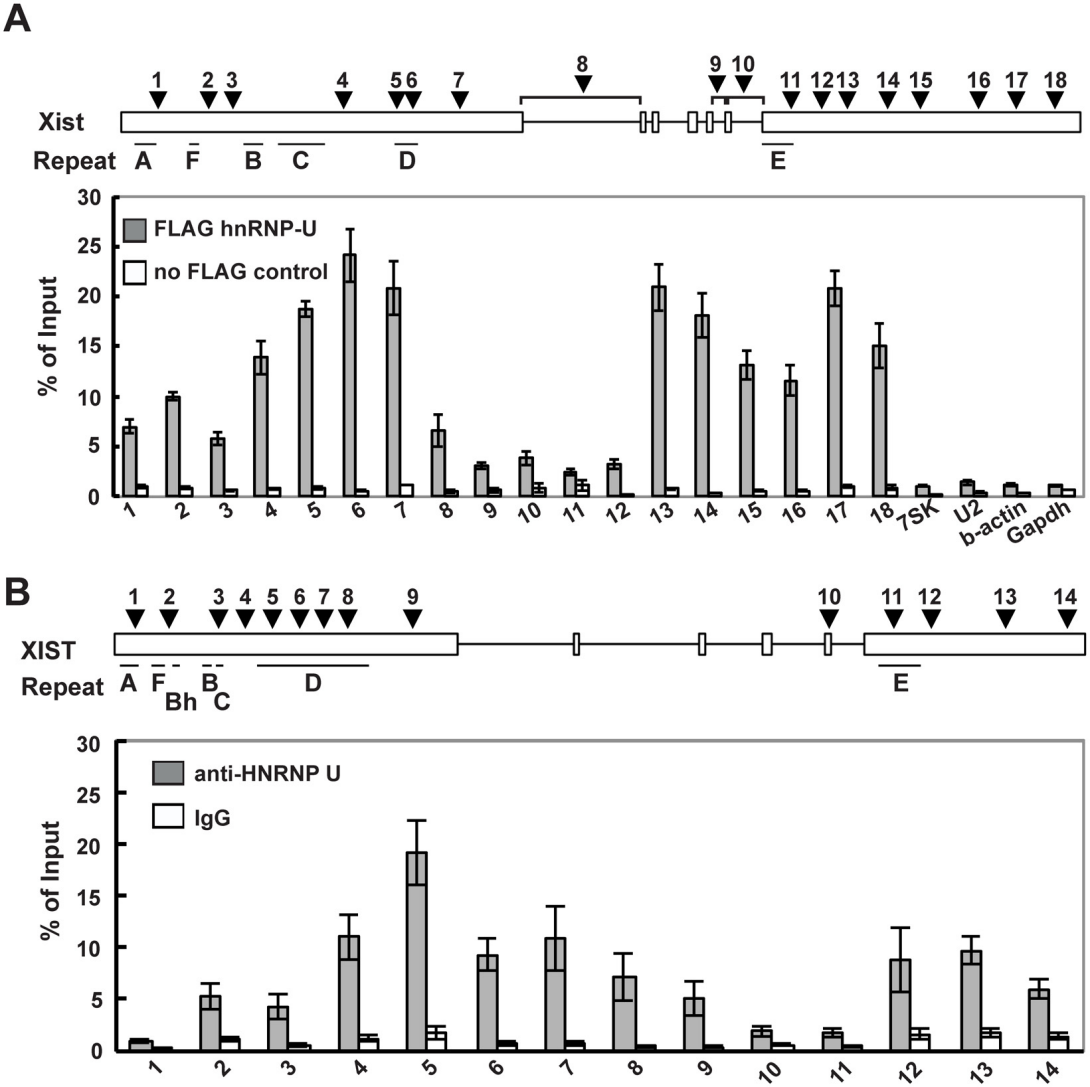
hnRNP U interacts directly with the first and last exons of mouse and human Xist RNA. (A) UV-crosslinking RIP analysis for Xist RNA and hnRNP U in mouse Neuro2A cells. A relative amount of each immunoprecipitated Xist RNA to input was quantified by RT-qPCR. The positions of the primer pairs are shown as arrowheads. The *Xist*-specific repeats (A-F) defined by Brockdorff et al. [51] are shown. (B) A relative amount of each immunoprecipitated Xist RNA to input was quantified by RT-qPCR. The positions of primer pairs are shown as arrowheads. The mean ± SD from four independent experiments is shown.

We previously reported that the nuclear matrix protein hnRNP U directly interacts with the Xist RNA via the RGG RNA-binding domain and is involved in the chromosomal localization of Xist RNA along the entire Xi region [35]. To further investigate the interaction between the Xist RNA and the hnRNP U protein across the *Xist* gene in detail, especially in exon 7 of Xist RNA, UV-crosslinking RNA-immunoprecipitation (UV-crosslinking RIP) analysis was carried out using Neuro2a cells as previously reported (Fig 4A). Multiple primer pairs were designed for the quantitative analysis to examine the binding affinity of hnRNP U to specific regions of the Xist RNA. The UV-crosslinking RIP showed that exons 1 and 7 of the Xist RNA could be significantly co-immunoprecipitated with the FLAG-hnRNP U, unlike the control 7SK, U2, beta-actin and Gapdh RNA. Within exon 1 of the Xist RNA, the second half of the exon (primer positions 4–7) exhibited preferential binding with hnRNP U over the first half of the exon (primer positions 1–3). Interestingly, the hnRNP U also bound to exon 7 of the Xist RNA with as high affinity at primer positions 13–18, with the exception of the repeat E region at primer positions 10–12. We also explored how hnRNP U interacted with the XIST RNA in human cells. Consistent with the data showing that the knockdown of human hnRNP U by siRNA induces the dissociation of XIST RNA from the Xi [43], we confirmed that the hnRNP U knockdown in human HEK293T cells led to the disperse localization of histone macroH2A, which accumulates on the Xi in an Xist RNA-dependent manner (S4 Fig) [19]. These data suggest that hnRNP U is essential for XIST RNA localization on the Xi in both humans and mice. Similar to the interaction between mouse Xist RNA and hnRNP U, the UV-crosslinking RIP analysis revealed that the human hnRNP U preferentially bound to XIST RNA broadly in the second half of the first exon and the last exon, except for the repeat E region (Fig 4B). Overall, our results suggest that exon 7 of the Xist RNA could contribute to the stable localization of Xist RNA on the Xi through interaction with hnRNP U.

### The *Xist* exon 7 deletion alters the interaction between Xist RNA and hnRNP U

**Fig 5:**
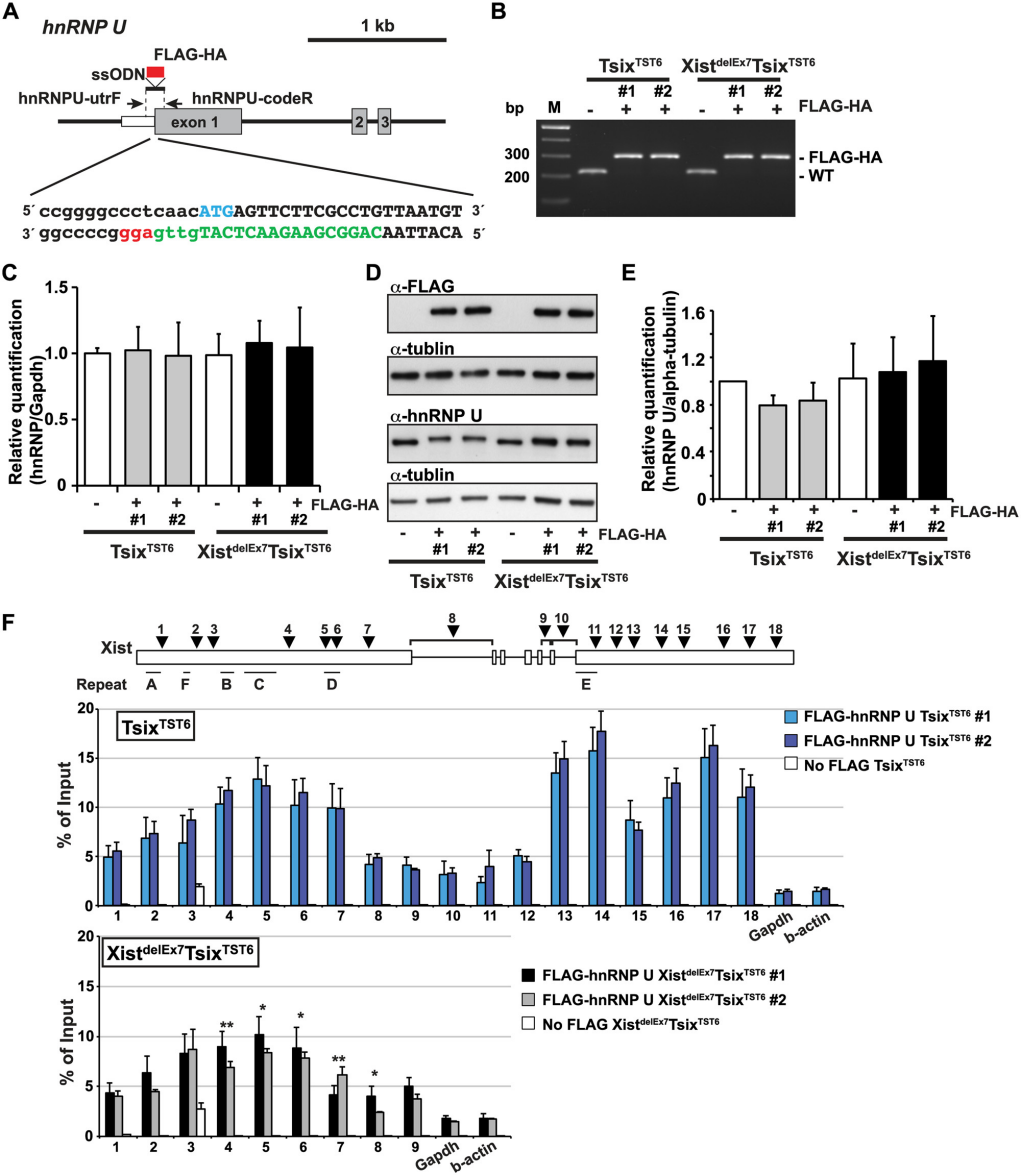
The exon 7 truncation in Xist RNA impairs the interaction between hnRNP U and Xist RNA. (A) Overview showing the generation of the FLAG-HA knock-in hnRNP U allele. The protein coding or 5´ UTR regions are shown as a gray or white box, respectively. The FLAG-HA tag is shown as a red box. The first ATG sequence of the translation start is labeled in blue. The protein coding or 5´ UTR regions are capitalized or lowercased, respectively. The sgRNA sequence is labeled in green. The protospacer-adjacent motif (PAM) sequence is labeled in red. (B) PCR genotyping analysis of the FLAG-HA tag knock-in hnRNP U allele. Genomic PCR using primers hnRNPU-utrF and hnRNPU-codeR amplified 212 or 284 bp PCR products from the wild-type or the FLAG-HA knock-in alleles, respectively. All FLAG-HA targeted knock-in ES cells shown in this work are homozygous knock-in clones. (C) Quantitative RT-PCR analysis of FLAGHA-hnRNP U using hnRNPU-F and hnRNPU-R in the parental and targeted ES cell lines at day 12 upon differentiation. The mean ± SEM from three independent experiments is shown. (D) A representative western blotting analysis using anti-FLAG, hnRNP U and tubulin antibodies with the parental and FLAG-HA hnRNP U knock-in ES clones at day 12 upon differentiation. (E) Quantification analysis of the hnRNP U western blotting, including Fig 5D. Each was normalized to the parental TST6 cells (set to 1). The mean ± SD values from three independent experiments are shown. (F) UV-crosslinking RIP analysis using Tsix^TST6^ and Xist^delEx7^Tsix^TST6^ mutant ES cell lines expressing FLAG-HA-tagged hnRNP U upon differentiation at day 12. The mean ± SD bar from three independent experiments is shown with an unpaired t test *P* values (*p<0.05, **p<0.01).

Next, to investigate whether the unstable Xist RNA localization and X-linked gene silencing on the Xi observed in the Xist^delEx7^Tsix^TST6^ mutant cells was due to the disturbed interaction between hnRNP U and the Xist RNA lacking exon 7, we performed UV-crosslinked RIP using Tsix^TST6^ and Xist^delEx7^Tsix^TST6^ mutant ES cell lines expressing a FLAG-HA epitope tag hnRNP U. To establish the ES cell lines expressing the FLAG-HA tagged hnRNP U from the endogenous hnRNP U loci, we used the CRISPR/Cas system. With this system, we obtained 2 and 3 FLAG-HA biallelic knock-in ES cell lines derived from the Tsix^TST6^ and Xist^delEx7^Tsix^TST6^ mutant ES cells, respectively (Fig 5A and 5B). By RT-qPCR and western blotting using anti-FLAG and anti-hnRNP U antibodies, we confirmed that the FLAG-HA knock-in ES cell lines expressed similar levels of FLAG-HA-hnRNP U to those of the parental ES cell lines (the Tsix^TST6^ and Xist^delEx7^Tsix^TST6^ mutant ES cell lines) on day 12 upon differentiation (Fig 5C–5E). We also confirmed that the FLAG-HA knock-in at the endogenous hnRNP U did not alter the kinetics of *Xist* expression upon differentiation and X-linked gene silencing compared to the parental ES cell lines (Figs 1D and 3A, S5A and S5B Fig). Furthermore, upon differentiation on day 12, the Tsix^TST6^ and Xist^delEx7^Tsix^TST6^ mutant EB cells expressing the FLAG-HA-hnRNP U from the endogenous loci exhibited a similar proportion of Xist RNA- and H3K27me3-positive cells to those observed in their parental cell lines (Fig 2 and S5C Fig).

Using EB cells expressing FLAGHA-hnRNP U at day 12 upon differentiation, UV-crosslinking RIP analysis followed by quantitative RT-PCR of the Xist RNA was carried out (Fig 5F). Tsix^TST6^ cells expressing wildtype Xist RNA exhibited a similar pattern of hnRNP U binding across Xist RNA, as observed in the Neuro2A cells showing preferential binding of hnRNP U to the 3´ regions of exon 1 and exon 7 of Xist RNA (with the exception of repeat E) (Fig 4A). Approximately 2.5–18% of the wild-type Xist RNA was precipitated by FLAG-hnRNP U immunoprecipitation at all primer pair positions in the differentiating EB of the Tsix^TST6^ and Xist^delEx7^Tsix^TST6^ mutant ES cell lines, while approximately 2–25% of the Xist RNA was recovered by FLAG-hnRNP U IP at a similar position in the Neuro2A cells (Figs 4A and 5E). The slightly lower Xist RIP efficiency that was observed in the EB cells could be due to the incomplete induction of X-inactivation (less than 80% on day 12) (Fig 2B) or to insufficient UV-irradiation on the inside of the sphere-shaped differentiating EB cells. Interestingly, the prominent binding of FLAG-HA-hnRNP U at the 3' region of exon 1 in Xist RNA was impaired by ~30% in the Xist^delEx7^Tsix^TST6^ mutant cells compared to that of the wild-type Xist RNA in the Tsix^TST6^ cells (Fig 5F). These data indicate that exon 7 of Xist RNA could be required for the interaction between the Xist RNA and hnRNP U or maintenance of the Xist RNA-hnRNP U RNP complex. Overall, these data suggest that exon 7 of Xist RNA is essential for stable Xist RNA localization and X-linked gene silencing, possibly acting through an interaction with hnRNP U.

## Discussion

In this study, we provided novel evidence that *Xist* exon 7 is necessary for the localization of Xist RNA during X-inactivation. Our results show that the aberrant localization of mutant Xist RNA lacking exon 7 on the Xi leads to the loss of the Xist cloud and the H3K27me3 modification on the Xi and impairs X-linked gene silencing due to the impaired binding affinity of Xist RNA to hnRNP U which bridges the Xist RNA and the Xi.

Previously, to examine the effects of *Xist* mutations on chromosome silencing and coating, tetracycline (tet)-inducible mutant Xist RNA constructs containing exon 7 specific deletions were generated and introduced into the *Hprt* locus of male ES cells [23]. The results showed that a large deletion of the 3' end of the Xist RNA that included exon 7 resulted in weak defects in Xist RNA localization on the Xi, X-linked gene silencing and MCB formation. In contrast to these results, our *Xist* truncation mutant at the endogenous locus exhibited a severe phenotype in terms of X-inactivation (Figs 2 and 3). Exon 7 truncated Xist RNA lost its ability to localize to the Xi and failed to induce or maintain X-linked gene silencing. Similar phenotypic differences were observed in the repeat C study in the Xist RNA [23,32,33]. Two independent studies using antisense PNA or LNA oligonucleotides to block the repeat C function of the Xist RNA revealed a crucial function of the repeat C in Xist RNA for its localization to the Xi [32,33]. On the other hand, a tet-inducible *Xist* transgene assay showed that the repeat C deletion in the Xist RNA affects neither the Xist RNA localization on the Xi nor the silencing of adjacent selection markers [23]. These differences might reflect differences in the systems used to elucidate the functional domain of the Xist RNA. One possibility is that the tet-inducible promoter used in the tet-inducible transgene assay might induce much higher levels of Xist expression [23]. Therefore, an abundant mutant Xist RNA could compensate for the defects of the mutant Xist RNA in the Xist RNA localization and X-linked gene silencing.

Interestingly, the tet-inducible *XIST* transgene assay in human cells showed that XIST RNA lacking a large part of its 3' region (from the end of the exon 1 through the last exon) displayed dispersed localization of XIST RNA on the targeted locus, yet could still induce adjacent EGFP silencing [50]. Furthermore, the deletion of the central region of XIST exon 1, including repeat C in the XIST transgene, did not affect the XIST localization [50]. Whereas human XIST RNA has only one poorly conserved repeat C in exon 1, there are 14 copies of a 120 bp repeat C in mice, which are crucial for Xist RNA function [32,33,51]. Thus, while both the first and last exons of Xist RNA are indispensable for its localization onto the Xi in mice, it is possible that the role of the second largest exon in human *XIST*, exon 6, might be more crucial for XIST RNA localization on the Xi than exon 1 in human cells.

To further explore the detailed mechanisms of how Xist RNA is stably anchored onto the Xi, we extended our previous approach [35] to clarify the detailed interaction between Xist RNA and hnRNP U. Because hnRNP U has an RGG RNA-binding domain required for the interaction with Xist RNA and a SAF domain for SAR/MAR attachment and DNA binding [40], it is likely that hnRNP U anchors Xist RNA onto the Xi to induce and maintain X-linked gene silencing during X-inactivation. The UV-crosslinking RIP analysis using a growing number of primers across the *Xist* gene showed that hnRNP U broadly bound not only to exon 1 of Xist RNA but also to exon 7 (Figs 4A and 5F). We also showed that the truncation mutation of Xist RNA lacking exon 7 results in an impaired interaction of the mutant Xist RNA with hnRNP U, indicating that exon 7 of Xist RNA is essential for the stable interaction with hnRNP U (Fig 5F). These results raise the possibility that stable Xist RNA localization on the Xi is mediated through the binding of hnRNP U to both exon 1 and 7 of the Xist RNA.

Previous work has shown that an absence of hnRNP U leads to the loss of the L-isoform of Xist RNA which contains the full length of exon 7, suggesting that an interaction of hnRNP to exon 7 of Xist RNA might regulate the stability of L-isoform of Xist RNA [35]. These observations suggest that the interaction between hnRNP U and exon 7 of Xist RNA is crucial for both the stability of Xist RNA and the localization of the Xist RNA on the Xi. Alternatively, hnRNP U might regulate the splicing of Xist RNA because hnRNP U is known to regulate the global control of alternative splicing of noncoding RNAs [52]. Although hnRNP U might regulate the splicing of nascent Xist RNAs transcribed at the endogenous *Xist* locus, it is unlikely that the hnRNP U accumulated across the entire Xi plays a role in the splicing of Xist RNA because the majority of Xist RNA is the spliced mature RNA [35]. Furthermore, a previous report has shown that even without splicing, inducible *Xist* cDNA transgenes can induce hnRNP U and Xist RNA localization to the Xi following gene silencing [36], indicating that Xist RNA-dependent hnRNP U accumulation on the Xi is not related to the splicing of Xist RNA. On the other hand, because the S-isoform of Xist RNA lacks a large region of exon 7 except for a 1.7 kb repeat E and 0.4 kb region of its 3´ end, it lacks a large hnRNP U binding region similar to the mutant Xist^delEx7^ RNA lacking the entire exon 7. It would be interesting to delineate the distinct roles of the L- and S-isoforms of Xist RNA in X-inactivation.

How does exon 7 of Xist RNA contribute to the stable interaction with hnRNP U and localization of Xist RNA on the Xi? One possibility is that multiple hnRNP U binding across exons 1 and 7 of Xist RNA might be essential for the stable localization of Xist RNA on the Xi. Thus, the mutant Xist^delEx7^ RNA lacking multiple hnRNP U binding sites within exon 7 of Xist RNA results in its unstable localization on the Xi. Alternatively, exon 7 of the Xist RNA might contribute to the stable localization of Xist RNA on the Xi through the RNP complex formation with hnRNP U. Because it is known that hnRNP U can form nucleic acid-mediated aggregation [53], multiple hnRNP U binding to both exon 1 and exon 7 within one Xist RNA molecule might facilitate the formation of an aggregated hnRNP U complex and stabilize the functional RNP complex. Without exon 7 of the Xist RNA, an insufficient number of hnRNP U might not be able to maintain the aggregated hnRNP U-Xist RNA complex, resulting in inefficient Xist RNA binding to the Xi.

To date, many chromatin modification enzymes have been known to interact with lncRNAs [54,55] but it remains unclear how these chromatin modification enzymes are recruited to their specific target loci to modify the chromatin structure. As a representative example, Xist RNA interacting with multiple chromatin modifying enzymes is targeted to the Xi to establish a multi-layer repressive epigenetic landscape [22]. While we note the potential role of hnRNP U to target Xist RNA to the Xi through interactions with specific RNA regions in this work, multiple recent reports have suggested that interactions between hnRNP proteins and lncRNAs play a pivotal role in numerous physiological processes, such as local gene regulation, nuclear organization, and the immune response [43,56–58]. Although the role of hnRNP proteins in each physiological process remains to be elucidated, these findings suggest that hnRNPs may be crucial players in the recruitment of lncRNA and its associated factors, such as chromatin modifying enzymes, onto their target loci. The interaction between Xist RNA and hnRNP U would be a great model to uncover how RNP complexes of lncRNAs, hnRNPs and chromatin-modifying enzymes are established and recruited to their specific target sites to regulate local gene regulation. Further studies of the interaction between the hnRNPs and lncRNAs will be necessary to shed light on the detailed molecular mechanism underlying the biological processes mediated by lncRNAs.

## Materials and Methods

### ES cells and cell culture

16.7 wild-type female ES cells [47] and their derivatives were maintained on irradiated male mouse embryonic fibroblast (MEF) feeder cells in Dulbecco´s modified Eagle´s medium (DMEM; Life Technologies) supplemented with 15% fetal bovine serum (FBS; Hyclone), 25 mM Hepes (pH 7.2–7.5) (Life Technologies), 1% MEM non-essential amino acid (Life Technologies), 1% GlutaMAX-I (Life Technologies), 100 units/ml Penicillin-Streptomycin (Life Technologies), 0.1 mM β-mercaptoethanol (Life Technologies), and 500 units/ml leukemia inhibitory factor (LIF). For the embryoid body (EB) differentiation [59], the ES cells were grown on feeder cells for 3 days and then partially trypsinized using 0.05% Trypsin-EDTA (Life Technologies). The trypsin reaction was stopped by adding differentiation media (ES media without LIF) and the ES cells were incubated for 30 min in a CO_2_ incubator to remove the feeder cells. The floating ES colonies were then transferred into bacterial plates and grown as a suspension culture in a CO_2_ incubator for the first 4 days. They were then attached onto gelatin-coated tissue culture plates.

### Targeted truncation of *Xist* and *Tsix*

The targeting vectors for the *Xist*/*Tsix* and *Tsix* truncation mutation were constructed using homologous recombination in bacteria [60]. A 9.3 kb XhoI-ClaI fragment from exon 2 to 7 of *Xist* from the sx9 P1 clone [61] was cloned into the XhoI-ClaI of pGEM7-DTAR [62], yielding pGEM-Xex2-7-DTAR. The pGEM-Xex2-7-DTAR was transfected to SW106 for recombination in bacteria. To generate the promoterless Hyg selection cassette, the BamHI fragment of SA-tpA from pSS-SAtpA1lox-DTAF [48] was subcloned into a pGEM vector derivative in which the EcoRI and HindIII sites in pGEM11-Zf(-) were replaced by SpeI, yielding pGEM-SAtpA-1. BamHI (Klenow-filled)-EcoRV Ires-Hyg fragment of pQCXIH (Clontech) was inserted into the Klenow-filled HindIII site between SA and tpA of pGEM-SAtpA-1 in the same direction. The BamHI SA-Ires-Hyg-tpA cassette was transferred into the pBluescript II SK- (pBS-SKII-) plasmid at the BamHI site, yielding pBS-SA-Ires-Hyg-tpA. To create a selection marker flanked by two FRT derivative F3 sites for the selection of homologous recombination in bacteria, the adaptor (F3-F and F3-R primers) was ligated into the HindIII/EcoRI of pBS-SKII- (pBS-2xF3), and a NheI-EcoRI zeocin (Zeo) cassette of pSKY [63] was cloned into the pBS-2xF3 at NheI and MfeI site between two F3 sites, yielding the pBS-2xF3-Zeo plasmid. For the construction of the *Tsix*-truncation targeting vector, the right arm (annealed XTST-F9L and XTST-R9L primers) for homologous recombination in bacteria was inserted into the BstBI/SacI of pBS-2xF3-Zeo, yielding pBS-2xF3-R85. The SpeI (Klenow-filled)-NotI SA-Ires-Hyg-tpA of pBS-SA-Ires-Hyg-tpA and the left arm (annealed TST-F8 and TST-R8 primers) were inserted into the HindIII (Klenow-filled)-XhoI of pBS-2xF3-Zeo-R85. tpA was replaced by an Eco53kI-NotI beta-actin pA (bpA) fragment of pGEM11-DTAL. For the *Xist*/*Tsix* truncation, the SA-tpA cassette was cloned into pBS-SKII(-). SA was replaced with an additional tpA fragment which was amplified with pA-BamXmaI-F and pA-BglII-R, yielding pBS-2xtpA-F. The left arm of the *Tsix*-truncation targeting construct for bacterial recombination was replaced with the left arm for *Xist*/*Tsix*-truncation (adaptor with XST-F7 and XST-R7) and EcoRV-NotI 2xtpA cassette from pBS-2xtpA-F. Selection cassettes with left and right homology arms for bacterial recombination were released by SalI-Bsu36I digestion and inserted into pGEM-Xex2-7-DTAR at position chrX: 103,468,781 and chrX: 103,468,781–103,468,821 in GRCm38/mm10 (UCSC genome browser) for the *Tsix*- and *Xist*/*Tsix*-truncation constructs, respectively. The Zeo selection marker was finally removed from the targeting constructs for recombination in bacteria by arabinose-inducible Flpe recombinase in the SW105 E. coli. The *Tsix*- and *Xist*/*Tsix*-truncation vector was linearized by XhoI digestion for homologous recombination in the ES cells.

ES cell targeting was performed as described previously [47]. Briefly, an ES cell suspension in ice-cold PBS with 3x10^6^ cells and 40 μg of the linearized targeting vector was used for electroporation using the BioRad GenePulser (240 V, 500 μF). 250 μg/ml hygromycin was added at 24 hours after transfection, and hygromycin-resistant colonies were picked on 8–9 days after electroporation. Colonies were screened by genomic PCR using 7.2-F and Hyg-F or SA-R and 7.2In-R primer pairs (S1 Fig and S1 Table).

### RT-qPCR and allele-specific RT–qPCR

The total RNA was isolated from undifferentiated ES and differentiating cells on days 0, 4, 8 and 12 upon differentiation by NucleoSpin RNA II (Clontech). A 2.5 μg of total RNA was converted into cDNA using Maxima H Minus reverse transcriptase (Thermo Scientific) according to the manufacturer's instructions. Real-time PCR was performed on Step One Plus real-time PCR System (Life Technologies) using the Fast SYBR Green Master Mix (Life Technologies). The primers and annealing temperature were: *Gapdh* (Gapdh-F and Gapdh-R; 60°C); *Nanog* (Nanog-F and Nanog-R; 60°C); *Oct3/4* (Oct3/4-F and Oct3/4-R; 62°C); *Xist* exon 1–3 (X1) for 129 allele (Xist^129^-E1-3-F and Xist^129^-E1-3-R; 62°C); *Xist* exon 7 (X2) for 129 allele (Xist^129^-E7-F and Xist^129^-E7-R; 58°C); *Tsix* intron 3–4 (T1) for 129 (Tsix^129^-In3-4-F and Tsix-In3-4-R; 58°C) and Cast (Tsix^Cast^-In3-4-F and Tsix-In3-4-R; 58°C) alleles; *Tsix* exon 4 (T2) for 129 (Tsix^129^-E4-F and Tsix^129^-E4-R) and Cast (Tsix^Cast^-E4-F and Tsix^129^-E4-R; 60°C) alleles; *Mecp2* for 129 allele (Mecp2^129^-F and Mecp2^129^-R; 58°C); *Pgk1* for 129 allele (Pgk1^129^-F and Pgk1^129^-R; 60°C). The ΔΔCt method was employed to analyze the relative changes in the gene expression levels from the real-time qPCR experiments, and Gapdh was used to normalize the data.

### Allele-specific RT-PCR

Allele-specific RT-PCR was performed as described [64]. Briefly, after the first strand synthesis described above, PCR was performed using Maxima Hot Start PCR Master Mix (Thermo Scientific), and an aliquot of the first round of PCR was cycled once in the second round of PCR. The PCR products were purified by ethanol precipitation, digested with restriction enzymes, transferred to Hybond N (GE Healthcare) and hybridized with a ^32^P-end-labeled oligonucleotide probe. The primers, probes and restriction enzymes were: *Xist* (XA-F and XA-R; XSP1 [62]; ScrFI); *Mecp2* (MeA-F and MeA-R; NS65 [62]; DdeI); *Pgk1* (Pgk1-F and Pgk1-R; Pgk1-P; MseI). Typhoon phosphorimager (GE Healthcare) was used to obtain quantitative measures. The data were quantified using GelEval (v1.37, Frog Dance Software) or ImageQuant (GE Healthcare).

### Half-life assay

The half-life assay for Xist RNA was performed with Click-iT Nascent RNA Capture Kit (Life Technologies) following the manufacturer’s instructions. Briefly, undifferentiated ES cells or EB were seeded to gelatinized 6-cm dishes and cultured with ES medium containing 0.1 mM 5-ethynyl uridine (EU) for 22 hrs. The next day, the EU-containing medium was replaced with EU-free medium, and cells were harvested at 0, 40, 80, 120, 180 and 240 min after changing the medium. 5 μg of total RNA extracted as above was biotinylated via the Click-iT reaction, precipitated by ethanol precipitation and dissolved in 50 μl of water. 1 μg of the biotinylated RNA was bound to Dynabeads MyOne Streptavidin T1 magnetic beads (Life Technologies) for 30 minutes at room temperature, and washed with 5x Click-iT reaction buffer 1 and 5x Click-iT reaction buffer 2. The biotinylated RNA-bound beads were resuspended with 12 μl of 5x Click-iT reaction buffer 2 and used as a template for RT-qPCR. RT-qPCR was performed as described above, with the 129 allele-specific primer set for *Xist* exon1-3 (X1), the S-isoform Xist specific primer set (XiI7SRT-F and XiI7SRT-R) or the L-isoform Xist specific primer set (XiI7LRT-F and XiI7LRT-R).

### Immuno-fluorescence in situ hybridization (Immuno-FISH) and immunofluorescence

Immuno-FISH was performed as described [65] with some modification. All ES and EB culture were dispersed by Accutase (Innovative Cell Technologies) and cyto-spinned onto slides at 1,500 rpm for 10 min. After Triton X-100 permeabilization in the cytoskeleton (CSK) buffer, the cells were fixed by 4% paraformaldehyde for 10 min at room temperature. An immunofluorescence assay using a mouse anti-H3K27me3 antibody (Active Motif, #61017) and an Alexa Fluor 555-labeled secondary antibody against mouse IgG (Life Technologies) was followed by 4% paraformaldehyde fixation for 10 min. RNA FISH was carried out with a 25 nM oligonucleotide probe cocktail, which hybridized from exon 1 to 6 of Xist RNA, in the hybridization buffer (10% formamide, 2x SSC, 2 mg/ml BSA, 10% dextran sulfate) at 37°C overnight. The next day, the slides were washed in wash buffer (10% formamide and 2x SSC), wash buffer with 5 ng/ml DAPI, and 2x SSC for 5 min each at 37°C. They were then treated with an antifade solution.

Immunofluorescence was carried out as described previously [35] using human HEK293T cells. Anti-human hnRNP-U (Santa Cruz, sc-32315) and anti-histone macroH2A (Upstate, 07–219) antibodies were used. The siRNA-mediated gene knockdown for human hnRNP U was performed with Silencer Select Pre-designed siRNA (siRNA ID: s6743, Ambion).

### UV-crosslinking RNA immunoprecipitation (RIP) analysis

UV-Crosslinking RIP was performed following a previously described procedure [35] with some modifications. Briefly, mouse Neuro2a or ES cells stably expressing Flag-tagged hnRNP-U or human HEK293T cells were rinsed with PBS and UV-irradiated with 400 mJ/cm^2^ at 254 nm. The cells were lysed in SDS buffer (50 mM Tris-HCl [pH 8.0], 1 mM EDTA, 150 mM NaCl, 1 mM DTT, 1% SDS, 1% Triton X-100) and incubated on ice for 10 min. Samples were sheared for 6 cycles of 30 sec ON at high power/30 sec OFF with the Bioruptor (Diagnode). Sonicated lysates were diluted 10-fold with dilution buffer (50 mM Tris-HCl [pH 8.0], 1 mM EDTA, 150 mM NaCl, 1 mM DTT, 1% Triton X-100, 1× protease inhibitor cocktail, RNase inhibitor). The soluble fraction was obtained by centrifuging at 15,000 rpm for 15 min at 4°C. One-tenth of the lysates for immunoprecipitation was kept as the “10% of input”. Anti-Flag M2 beads (Sigma), or protein G beads (Millipore) conjugated with 3 μg of control IgG (Jackson or Millipore) or anti-hnRNP-U (3G6, Santa Cruz) were added to the cell lysates and rotated at 4°C for 2 h. The beads were washed twice with high salt buffer (20 mM Tris-HCl [pH 8.0], 1 mM EDTA, 500 mM NaCl, 1 mM DTT, 0.1% SDS, 1% Triton X-100) and three times with low salt buffer (20 mM Tris-HCl [pH 8.0] 1 mM EDTA, 150 mM NaCl, 1 mM DTT, 0.1% SDS, 1% Triton X-100). The washed beads were treated with proteinase K at 37°C for 1 h, and the RNA was extracted with Trizol LS Reagent (Life Technologies) according to the manufacturer’s instructions. Purified RNA treated with DNase I was dissolved in 20 μl nuclease free water, and cDNA was synthesized using 2 μl of the RNA samples. Quantitative PCR was performed using the primers listed in S1 Table.

### Targeted knock-in of the FLAG-HA epitope tag to hnRNP U by the CRISPR/Cas system

pSpCas9(BB)-2A-Puro [66] (pX459, Addgene plasmid #48139) was used for CRISPR/Cas-mediated FLAG-HA knock-in to the 5´-end of the hnRNP U coding region. To avoid the premature termination of sgRNA and to improve the sgRNA-Cas9 assembly, mutations were introduced to pX459 [67]. pX459 was digested with BbsI and KpnI and ligated with three pairs of oligonucleotides (hnRNPU-CRI-F & hnRNPU-CRI-R, sgRNA-[F+E]-F1 & sgRNA-[F+E]-R1, sgRNA-[F+E]-F2 & sgRNA-[F+E]-R2), each of which were phosphorylated, denatured and annealed before ligation, yielding pX459FE-hnRNPU. 5x10^6^ ES cells were plated on 6-well plates with feeder cells 8 hours before transfection. Cells were transfected with FuGENE HD (Roche) according to the manufacturer’s instructions, with 1 μg pX459FE-hnRNPU vector and 1 μg single-stranded oligodeoxynucleotide (hnRNPU-FLAGHA-KI) for the homology directed repair (HDR)-mediated FLAG-HA knock-in. 16 hour after transfection, 2 μg/ml of puromycin was added to the ES media and ES cells were cultured for 24 hours. After puromycin selection, cells were trypsinized and 1/5 of cells were plated onto a 10 cm dish with feeder cells without any selection drug. Colonies were picked on 8 days after plating.

### Western blotting

Differentiated EB cells were lysed on day 12 with RIPA buffer and sonicated using a Bioruptor (Diagenode) set on “HIGH” for 4 cycles of 30 sec ON and 30 sec OFF. Equal amounts of total protein (20 μg per lane) were applied and run on a 4–15% SDS-polyacrylamide gel and transferred to PVDF membranes (Millipore). Membranes were blocked by 5% skim milk in TBST (50 mM Tris-HCl [pH 7.5], 150 mM NaCl, 0.1% Tween-20) at room temperature for 1 h, and incubated using primary antibodies against FLAG M2 (mouse 1:2,000, Sigma), hnRNP U (clone 3G6, mouse 1:1,000, Millipore) and alpha Tubulin DM1A (mouse 1:5,000, Santa Cruz) overnight at 4°C. After incubation with the secondary antibody conjugated to horseradish peroxidase (HRP) (anti-mouse IgG 1:10,000, Jackson ImmunoResearch) at room temperature for 1 h, immune complexes were visualized using an enhanced chemiluminescence (ECL) detection system (Thermo Scientific) or Immobilon Western chemiluminescent HRP substrate (Millipore), and quantitated by AlphaView (Alpha Innotech).

## Supporting Information

S1 Fig Creation of the *Xist*/*Tsix* double truncation Xist^delE7^Tsix^TST6^ and *Tsix* truncation Tsix^TST6^ mutant female ES cells. (A) Map of the mouse *Xist*/*Tsix* locus. Arrows indicate the position and direction of the primers used. (B) Genomic PCR analysis to confirm FLAG-HA knock-in.
(EPS)

S2 Fig Half-life assay for the S- and L-isoforms of Xist RNA on day 8 upon differentiation. (A) A map shows alternative splicing of *Xist* with the 3´ end of the Xist^delEx7^ mutant RNA. (B) RT-qPCR was performed using the S-isoform-specific (XiI7SRT-F and XiI7SRT-R) and L-isoform-specific primer sets (XiI7LRT-F and XiI7LRT-R). The mean ± SD values from two independent experiments are shown.
(EPS)

S3 Fig The *Xist*/*Tsix* double truncation Xist^delE7^Tsix^TST6^ and *Tsix* truncation Tsix^TST6^ mutations do not affect EB differentiation. (A) RT-qPCR analysis for the expression of pluripotent markers (Nanog and Oct3/4). Each Nanog and Oct3/4 expression level was normalized to that of differentiation day 0 (set to 1) and Gapdh, which was used as an internal control. The mean ± SD bar for three independent experiments is shown. (B) Representative phase contrast images of the differentiated EB cells on differentiation at day 8.
(EPS)

S4 Fig hnRNP U knockdown results in the loss of histone macroH2A localization on the Xi in human cells. Immunofluorescence of hnRNP U (magenta) and histone macroH2A (green) in the control and hnRNP U knockdown HEK293T cells. The arrowhead indicates a hnRNP U knockdown cell.
(EPS)

S5 Fig FLAG-HA knock-in at the endogenous hnRNP U loci by CRISPR-Cas does not affect X-inactivation. (A) 129 allele-specific RT-qPCR analysis of Xist expression in the FLAG-HA targeted knock-in ES cells using primers that extend through *Xist* exons 1 to 3. The expression value was normalized to that of Tsix^TST6^ at day 0 (set to 1,) and Gapdh was used as an internal control. The graph shows the mean ± SD bar from three independent experiments. (B) 129 allele-specific RT-qPCR analysis of the X-linked *Pgk1* gene upon differentiation, normalized to undifferentiated cells (set to 1), with Gapdh used as an internal control. The mean ± SD bar from three independent experiments is shown. *P* values were calculated using an unpaired t test (*p<0.05, **p<0.01, ***p<0.001). (C) Top: Immuno-FISH for Xist RNA (green) and H3K27me3 (red) in FLAG-HA targeted knock-in ES cells after differentiation (day 12). Nuclei were counterstained with DAPI. Scale bar, 10 μm. Bottom: frequency of the Xist cloud- and H3K27me3-positive cells on day 12 upon differentiation. More than 300 nuclei in each ES cell line at each time point from three independent experiments were counted and classified based on the Xist RNA and H3K27me3 signal.
(AI) Click here for additional data file.

S1 Table The list of primers used in this study. (DOCX)

## References

[1] Carninci P, Kasukawa T, Katayama S, Gough J, Frith MC, et al (2005) The transcriptional landscape of the mammalian genome. Science 309: 1559–1563. 16141072 10.1126/science.1112014

[2] Mattick JS, Makunin IV (2006) Non-coding RNA. Hum Mol Genet 15 Spec No 1: R17–29. 16651366 10.1093/hmg/ddl046

[3] Mercer TR, Mattick JS (2013) Structure and function of long noncoding RNAs in epigenetic regulation. Nat Struct Mol Biol 20: 300–307. 10.1038/nsmb.2480 23463315

[4] Rinn JL, Chang HY (2012) Genome regulation by long noncoding RNAs. Annu Rev Biochem 81: 145–166. 10.1146/annurev-biochem-051410-092902 22663078 PMC3858397

[5] Kitagawa M, Kitagawa K, Kotake Y, Niida H, Ohhata T (2013) Cell cycle regulation by long non-coding RNAs. Cell Mol Life Sci 70: 4785–4794. 10.1007/s00018-013-1423-0 23880895 PMC3830198

[6] Fatica A, Bozzoni I (2014) Long non-coding RNAs: new players in cell differentiation and development. Nat Rev Genet 15: 7–21. 10.1038/nrg3606 24296535

[7] Kornfeld JW, Bruning JC (2014) Regulation of metabolism by long, non-coding RNAs. Front Genet 5: 57 10.3389/fgene.2014.00057 24723937 PMC3971185

[8] Cheetham SW, Gruhl F, Mattick JS, Dinger ME (2013) Long noncoding RNAs and the genetics of cancer. Br J Cancer 108: 2419–2425. 10.1038/bjc.2013.233 23660942 PMC3694235

[9] Gutschner T, Diederichs S (2012) The hallmarks of cancer: a long non-coding RNA point of view. RNA Biol 9: 703–719. 10.4161/rna.20481 22664915 PMC3495743

[10] Payer B, Lee JT (2008) X chromosome dosage compensation: how mammals keep the balance. Annu Rev Genet 42: 733–772. 10.1146/annurev.genet.42.110807.091711 18729722

[11] Maclary E, Hinten M, Harris C, Kalantry S (2013) Long nonoding RNAs in the X-inactivation center. Chromosome Res 21: 601–614. 10.1007/s10577-013-9396-2 24297756 PMC3919162

[12] Augui S, Nora EP, Heard E (2011) Regulation of X-chromosome inactivation by the X-inactivation centre. Nat Rev Genet 12: 429–442. 10.1038/nrg2987 21587299

[13] Penny GD, Kay GF, Sheardown SA, Rastan S, Brockdorff N (1996) Requirement for Xist in X chromosome inactivation. Nature 379: 131–137. 8538762 10.1038/379131a0

[14] Clemson CM, McNeil JA, Willard HF, Lawrence JB (1996) XIST RNA paints the inactive X chromosome at interphase: evidence for a novel RNA involved in nuclear/chromosome structure. J Cell Biol 132: 259–275. 8636206 10.1083/jcb.132.3.259 PMC2120729

[15] Plath K, Fang J, Mlynarczyk-Evans SK, Cao R, Worringer KA, et al (2003) Role of histone H3 lysine 27 methylation in X inactivation. Science 300: 131–135. 12649488 10.1126/science.1084274

[16] Silva J, Mak W, Zvetkova I, Appanah R, Nesterova TB, et al (2003) Establishment of histone h3 methylation on the inactive X chromosome requires transient recruitment of Eed-Enx1 polycomb group complexes. Dev Cell 4: 481–495. 12689588 10.1016/s1534-5807(03)00068-6

[17] Csankovszki G, Nagy A, Jaenisch R (2001) Synergism of Xist RNA, DNA methylation, and histone hypoacetylation in maintaining X chromosome inactivation. J Cell Biol 153: 773–784. 11352938 10.1083/jcb.153.4.773 PMC2192370

[18] Brown CJ, Willard HF (1994) The human X-inactivation centre is not required for maintenance of X-chromosome inactivation. Nature 368: 154–156. 8139659 10.1038/368154a0

[19] Csankovszki G, Panning B, Bates B, Pehrson JR, Jaenisch R (1999) Conditional deletion of Xist disrupts histone macroH2A localization but not maintenance of X inactivation. Nat Genet 22: 323–324. 10431231 10.1038/11887

[20] Zhang LF, Huynh KD, Lee JT (2007) Perinucleolar targeting of the inactive X during S phase: evidence for a role in the maintenance of silencing. Cell 129: 693–706. 17512404 10.1016/j.cell.2007.03.036

[21] Yildirim E, Kirby JE, Brown DE, Mercier FE, Sadreyev RI, et al (2013) Xist RNA is a potent suppressor of hematologic cancer in mice. Cell 152: 727–742. 10.1016/j.cell.2013.01.034 23415223 PMC3875356

[22] Sado T, Brockdorff N (2013) Advances in understanding chromosome silencing by the long non-coding RNA Xist. Philos Trans R Soc Lond B Biol Sci 368: 20110325 10.1098/rstb.2011.0325 23166390 PMC3539355

[23] Wutz A, Rasmussen TP, Jaenisch R (2002) Chromosomal silencing and localization are mediated by different domains of Xist RNA. Nat Genet 30: 167–174. 11780141 10.1038/ng820

[24] Zhao J, Sun BK, Erwin JA, Song JJ, Lee JT (2008) Polycomb proteins targeted by a short repeat RNA to the mouse X chromosome. Science 322: 750–756. 10.1126/science.1163045 18974356 PMC2748911

[25] Davidovich C, Wang X, Cifuentes-Rojas C, Goodrich KJ, Gooding AR, et al (2015) Toward a consensus on the binding specificity and promiscuity of PRC2 for RNA. Mol Cell 57: 552–558. 10.1016/j.molcel.2014.12.017 25601759 PMC4320675

[26] Davidovich C, Zheng L, Goodrich KJ, Cech TR (2013) Promiscuous RNA binding by Polycomb repressive complex 2. Nat Struct Mol Biol 20: 1250–1257. 10.1038/nsmb.2679 24077223 PMC3823624

[27] Zhao J, Ohsumi TK, Kung JT, Ogawa Y, Grau DJ, et al (2010) Genome-wide identification of polycomb-associated RNAs by RIP-seq. Mol Cell 40: 939–953. 10.1016/j.molcel.2010.12.011 21172659 PMC3021903

[28] Kaneko S, Son J, Shen SS, Reinberg D, Bonasio R (2013) PRC2 binds active promoters and contacts nascent RNAs in embryonic stem cells. Nat Struct Mol Biol 20: 1258–1264. 10.1038/nsmb.2700 24141703 PMC3839660

[29] Kanhere A, Viiri K, Araujo CC, Rasaiyaah J, Bouwman RD, et al (2010) Short RNAs are transcribed from repressed polycomb target genes and interact with polycomb repressive complex-2. Mol Cell 38: 675–688. 10.1016/j.molcel.2010.03.019 20542000 PMC2886029

[30] Khalil AM, Guttman M, Huarte M, Garber M, Raj A, et al (2009) Many human large intergenic noncoding RNAs associate with chromatin-modifying complexes and affect gene expression. Proc Natl Acad Sci U S A 106: 11667–11672. 10.1073/pnas.0904715106 19571010 PMC2704857

[31] Cifuentes-Rojas C, Hernandez AJ, Sarma K, Lee JT (2014) Regulatory interactions between RNA and polycomb repressive complex 2. Mol Cell 55: 171–185. 10.1016/j.molcel.2014.05.009 24882207 PMC4107928

[32] Beletskii A, Hong YK, Pehrson J, Egholm M, Strauss WM (2001) PNA interference mapping demonstrates functional domains in the noncoding RNA Xist. Proc Natl Acad Sci U S A 98: 9215–9220. 11481485 10.1073/pnas.161173098 PMC55400

[33] Sarma K, Levasseur P, Aristarkhov A, Lee JT (2010) Locked nucleic acids (LNAs) reveal sequence requirements and kinetics of Xist RNA localization to the X chromosome. Proc Natl Acad Sci U S A 107: 22196–22201. 10.1073/pnas.1009785107 21135235 PMC3009817

[34] Jeon Y, Lee JT (2011) YY1 tethers Xist RNA to the inactive X nucleation center. Cell 146: 119–133. 10.1016/j.cell.2011.06.026 21729784 PMC3150513

[35] Hasegawa Y, Brockdorff N, Kawano S, Tsutui K, Tsutui K, et al (2010) The matrix protein hnRNP U is required for chromosomal localization of Xist RNA. Dev Cell 19: 469–476. 10.1016/j.devcel.2010.08.006 20833368

[36] Pullirsch D, Hartel R, Kishimoto H, Leeb M, Steiner G, et al (2010) The Trithorax group protein Ash2l and Saf-A are recruited to the inactive X chromosome at the onset of stable X inactivation. Development 137: 935–943. 10.1242/dev.035956 20150277 PMC2834461

[37] Romig H, Fackelmayer FO, Renz A, Ramsperger U, Richter A (1992) Characterization of SAF-A, a novel nuclear DNA binding protein from HeLa cells with high affinity for nuclear matrix/scaffold attachment DNA elements. EMBO J 11: 3431–3440. 1324173 10.1002/j.1460-2075.1992.tb05422.x PMC556878

[38] Kiledjian M, Dreyfuss G (1992) Primary structure and binding activity of the hnRNP U protein: binding RNA through RGG box. EMBO J 11: 2655–2664. 1628625 10.1002/j.1460-2075.1992.tb05331.x PMC556741

[39] Tsutsui K, Tsutsui K, Okada S, Watarai S, Seki S, et al (1993) Identification and characterization of a nuclear scaffold protein that binds the matrix attachment region DNA. J Biol Chem 268: 12886–12894. 8509422

[40] Kim MK, Nikodem VM (1999) hnRNP U inhibits carboxy-terminal domain phosphorylation by TFIIH and represses RNA polymerase II elongation. Mol Cell Biol 19: 6833–6844. 10490622 10.1128/mcb.19.10.6833 PMC84680

[41] Kukalev A, Nord Y, Palmberg C, Bergman T, Percipalle P (2005) Actin and hnRNP U cooperate for productive transcription by RNA polymerase II. Nat Struct Mol Biol 12: 238–244. 15711563 10.1038/nsmb904

[42] Fu D, Collins K (2007) Purification of human telomerase complexes identifies factors involved in telomerase biogenesis and telomere length regulation. Mol Cell 28: 773–785. 18082603 10.1016/j.molcel.2007.09.023 PMC2917595

[43] Hacisuleyman E, Goff LA, Trapnell C, Williams A, Henao-Mejia J, et al (2014) Topological organization of multichromosomal regions by the long intergenic noncoding RNA Firre. Nat Struct Mol Biol 21: 198–206. 10.1038/nsmb.2764 24463464 PMC3950333

[44] Kipp M, Gohring F, Ostendorp T, van Drunen CM, van Driel R, et al (2000) SAF-Box, a conserved protein domain that specifically recognizes scaffold attachment region DNA. Mol Cell Biol 20: 7480–7489. 11003645 10.1128/mcb.20.20.7480-7489.2000 PMC86301

[45] Helbig R, Fackelmayer FO (2003) Scaffold attachment factor A (SAF-A) is concentrated in inactive X chromosome territories through its RGG domain. Chromosoma 112: 173–182. 14608463 10.1007/s00412-003-0258-0

[46] Yen ZC, Meyer IM, Karalic S, Brown CJ (2007) A cross-species comparison of X-chromosome inactivation in Eutheria. Genomics 90: 453–463. 17728098 10.1016/j.ygeno.2007.07.002

[47] Lee JT, Lu N (1999) Targeted mutagenesis of Tsix leads to nonrandom X inactivation. Cell 99: 47–57. 10520993 10.1016/s0092-8674(00)80061-6

[48] Ogawa Y, Sun BK, Lee JT (2008) Intersection of the RNA interference and X-inactivation pathways. Science 320: 1336–1341. 10.1126/science.1157676 18535243 PMC2584363

[49] Sun BK, Deaton AM, Lee JT (2006) A transient heterochromatic state in Xist preempts X inactivation choice without RNA stabilization. Mol Cell 21: 617–628. 16507360 10.1016/j.molcel.2006.01.028

[50] Chow JC, Hall LL, Baldry SE, Thorogood NP, Lawrence JB, et al (2007) Inducible XIST-dependent X-chromosome inactivation in human somatic cells is reversible. Proc Natl Acad Sci U S A 104: 10104–10109. 17537922 10.1073/pnas.0610946104 PMC1891207

[51] Brockdorff N, Ashworth A, Kay GF, McCabe VM, Norris DP, et al (1992) The product of the mouse Xist gene is a 15 kb inactive X-specific transcript containing no conserved ORF and located in the nucleus. Cell 71: 515–526. 1423610 10.1016/0092-8674(92)90519-i

[52] Xiao R, Tang P, Yang B, Huang J, Zhou Y, et al (2012) Nuclear matrix factor hnRNP U/SAF-A exerts a global control of alternative splicing by regulating U2 snRNP maturation. Mol Cell 45: 656–668. 10.1016/j.molcel.2012.01.009 22325991 PMC3299905

[53] Fackelmayer FO, Dahm K, Renz A, Ramsperger U, Richter A (1994) Nucleic-acid-binding properties of hnRNP-U/SAF-A, a nuclear-matrix protein which binds DNA and RNA in vivo and in vitro. Eur J Biochem 221: 749–757. 8174554 10.1111/j.1432-1033.1994.tb18788.x

[54] Koziol MJ, Rinn JL (2010) RNA traffic control of chromatin complexes. Curr Opin Genet Dev 20: 142–148. 10.1016/j.gde.2010.03.003 20362426 PMC2895502

[55] Wang KC, Chang HY (2011) Molecular mechanisms of long noncoding RNAs. Mol Cell 43: 904–914. 10.1016/j.molcel.2011.08.018 21925379 PMC3199020

[56] Huarte M, Guttman M, Feldser D, Garber M, Koziol MJ, et al (2010) A large intergenic noncoding RNA induced by p53 mediates global gene repression in the p53 response. Cell 142: 409–419. 10.1016/j.cell.2010.06.040 20673990 PMC2956184

[57] Carpenter S, Aiello D, Atianand MK, Ricci EP, Gandhi P, et al (2013) A long noncoding RNA mediates both activation and repression of immune response genes. Science 341: 789–792. 10.1126/science.1240925 23907535 PMC4376668

[58] Li Z, Chao TC, Chang KY, Lin N, Patil VS, et al (2014) The long noncoding RNA THRIL regulates TNFalpha expression through its interaction with hnRNPL. Proc Natl Acad Sci U S A 111: 1002–1007. 10.1073/pnas.1313768111 24371310 PMC3903238

[59] Martin GR, Evans MJ (1975) Differentiation of clonal lines of teratocarcinoma cells: formation of embryoid bodies in vitro. Proc Natl Acad Sci U S A 72: 1441–1445. 1055416 10.1073/pnas.72.4.1441 PMC432551

[60] Lee EC, Yu D, Martinez de Velasco J, Tessarollo L, Swing DA, et al (2001) A highly efficient Escherichia coli-based chromosome engineering system adapted for recombinogenic targeting and subcloning of BAC DNA. Genomics 73: 56–65. 11352566 10.1006/geno.2000.6451

[61] Erwin JA, del Rosario B, Payer B, Lee JT (2012) An ex vivo model for imprinting: mutually exclusive binding of Cdx2 and Oct4 as a switch for imprinted and random X-inactivation. Genetics 192: 857–868. 10.1534/genetics.112.144121 22942124 PMC3522163

[62] Ogawa Y, Lee JT (2003) Xite, X-inactivation intergenic transcription elements that regulate the probability of choice. Mol Cell 11: 731–743. 12667455 10.1016/s1097-2765(03)00063-7

[63] Yang Y, Seed B (2003) Site-specific gene targeting in mouse embryonic stem cells with intact bacterial artificial chromosomes. Nat Biotechnol 21: 447–451. 12627171 10.1038/nbt803

[64] Stavropoulos N, Lu N, Lee JT (2001) A functional role for Tsix transcription in blocking Xist RNA accumulation but not in X-chromosome choice. Proc Natl Acad Sci U S A 98: 10232–10237. 11481444 10.1073/pnas.171243598 PMC56944

[65] Yue M, Charles Richard JL, Yamada N, Ogawa A, Ogawa Y (2014) Quick fluorescent in situ hybridization protocol for Xist RNA combined with immunofluorescence of histone modification in X-chromosome inactivation. J Vis Exp: e52053 10.3791/52053 25489864 PMC4354415

[66] Ran FA, Hsu PD, Wright J, Agarwala V, Scott DA, et al (2013) Genome engineering using the CRISPR-Cas9 system. Nat Protoc 8: 2281–2308. 10.1038/nprot.2013.143 24157548 PMC3969860

[67] Chen B, Gilbert LA, Cimini BA, Schnitzbauer J, Zhang W, et al (2013) Dynamic imaging of genomic loci in living human cells by an optimized CRISPR/Cas system. Cell 155: 1479–1491. 10.1016/j.cell.2013.12.001 24360272 PMC3918502

