# Supplementary material for: Xist Exon 7 Contributes to the Stable Localization of Xist RNA on the Inactive X-Chromosome
Source: PLoS Genet. 2015 Aug 5;11(8):e1005430. doi: 10.1371/journal.pgen.1005430 (PMC4526699; doi:10.1371/journal.pgen.1005430)
Supplement: S1 Table — (DOCX) [file pgen.1005430.s006.docx]

**Table S1. The list of primers used in this study.**

**Tsix and Xist/Tsix truncation**

F3-F, agcttgaagttcctattcttcaaatagtataggaacttcgctagcgggcaattggggtacgtagaagttcctattcttcaaatagtataggaacttcg

F3-R, aattcgaagttcctatactatttgaagaataggaacttctacgtaccccaattgcccgctagcgaagttcctatactatttgaagaataggaacttca

XTST-F9L, cgaatttgctggaagatggtgctgggtggagagcatctaatgtgataatgtgaggcagggccatgtacacgatggaagatgaacaggcctaaggagct

XTST-R9L, ccttaggcctgttcatcttccatcgtgtacatggccctgcctcacattatcacattagatgctctccacccagcaccatcttccagcaaatt

TST-F8, tcgatcgatgtcgacttaaaagcaatagaatgaggtaagtcactagcattgcagtcttctgaggatttgc

TST-R8, ggccgcaaatcctcagaagactgcaatgctagtgacttacctcattctattgcttttaagtcgacatcgat

XST-F7, tcgactttataaatgacccgaggatcaacatgcctgactgcagcatcttaaaagcaatagaat

XST-R7, attctattgcttttaagatgctgcagtcaggcatgttgatcctcgggtcatttataaag

pA-BamXmaI-F, gttggatcccccgggatctgcgactctagaggatctgcga

pA-BglII-R, gccagatctcagacatgataagatacattgatgagtttggacaa

7.2F, gtctcagttgccttctccttgctcccactg

Hyg-F, atgcgcgattgctgatccccatgtgtatca

SA-R, aaaccctggactactgcgccctacagatct

7.2In-R, tcccagacctcttcaacctggctccatctt

**RT-qPCR and allele-specific RT-qPCR**

gene (allele); forward primer; reverse primer

Gapdh; Gapdh-F, tccatgacaactttggcattg; Gapdh-R, cagtcttctgggtggcagtga

Nanog; Nanog-F, tgctactgagatgctctgcac; Nanog-R, accactggtttttctgccac

Oct3/4; Oct3/4-F, tgaagttggagaaggtggaac; Oct3/4-R, tgtaccccaaggtgatcctc

Xist X1 (129); Xist-E1-3-F, ctgctcctccgttacatcag; Xist^129^-E1-3-R, ctgctggcagtccttgtg

Xist X2 (129); Xist-E7-F, gggaaaacaatgtcaggctatc; Xist^129^-E7-R, gctcacctaagcccaaagtaa

Tsix T1 (129); Tsix^129^-In3-4-F, ccgccatgtttggattact; Tsix-In3-4-R, ttctggggagtcagtgtttg

Tsix T1 (Cast); Tsix^Cast^-In3-4-F, cgccatgtttggattgac; Tsix-In3-4-R, ttctggggagtcagtgtttg

Tsix T2 (129); Tsix^129^-E4-F, tcttgcaaacctcatcattgtt; Tsix-E4-R, tatctctccagcccaggaac

Tsix T2 (Cast); Tsix^Cast^-E4-F, ttcttgcaaacctcatcattgta; Tsix-E4-R, tatctctccagcccaggaac

Mecp2 (129); Mecp2-F, cagcagcatctgcaaagaag; Mecp2^129^-R, tttctgcaactgtggtagtcgt

Pgk1 (129); Pgk1-F, gatgagggtggacttcaacg; Pgk1^129^-R, ccattgtccaagcagaatatg

Xist S-isoform; XiI7SRT-F, tccaagagcggggaactact; XiI7SRT-R, gttggtgggaagatgactcca

Xist L-isoform; XiI7LRT-F, ccagccagtgtcaccttctt; XiI7LRT-R, gcaccaaattactcggccac

**Allele-specific RT-PCR**

gene; forward primer; reverse primer

Xist; XA-F, ttggcagcaagtgcctttac; XA-R, tgctggcagtccttgagtct

Mecp2; MeA-F, tggtagctgggatgttaggg; MeA-R, gcaaggtggggtcatcatac

Pgk1; Pgk1-F, caagctgactttggacaagc; Pgk1-R, acaggcattctcgacttctg

probe

Pgk1-P, aatctctgctgggcaaggatgttctgttcttgaaggattg

**Crosslinking RNA-immunoprecipitation**

**mouse Neuro 2A Xist**

forward primer; reverse primer

1Fw, gtgtactgttgctgctg; 1Rv, gtatcacgcagaagcca

2Fw, gactgtgatgagtcactg; 2Rv, tccgacatcatccaacac

3Fw, cgtctgatagtgtgctttgcta; 3Rv, aagagtagctcggtggatgagt

4Fw, ctgttggcatgctgtcatg; 4Rv, aatgttccagtgcagagg

5Fw, ccaatacggtcaatggtcct; 5Rv, tgaggaaggggtttcaagtg

6Fw, tgcacacatctcattccatttg; 6Rv, ggggacagccttatccagtg

7Fw, atctttgcttggtctttactaca; 7Rv, aaaataagcaaggactggtgac

8Fw, cccgctgctgagtgtttgat; 8Rv, atccaggcaatccttcttcttg

9Fw, gaaagctttgccagctgttt; 9Rv, acccagttttctgtgctgct

10Fw, cccaaagcagcacagaaaact; 10Rv, cacacccacaatacacactcattcta

11Fw, gttgcatgcatccctctctt; 11Rv, cagagaaagtggcccaagaa

12Fw, caggacacctgtgacttcca; 12Rv, tccagagttctgggccatag

13Fw, ggaaagcatcaggttgttagg; 13Rv, cttctcagagggttggcacat

14Fw, ctgctttggtgaggctcagt; 14Rv, cccctttgttattcccagtg

15Fw, cagtgtgtccacacttgag; 15Rv, ttcagtggttcacagttac

16Fw, gagtacgctgttgctgtc; 16Rv, agagtctgactgctcttc

17Fw, tttgttccaaaagggcactc; 17Rv, acctctgaggcaaactgtgg

18Fw, gctgaagtcacaatttgctg; 18Rv, cttcaacctggctccatct

7SK Fw, gacgaccttccccgaatagag; 7SK Rv, gagcttgtttggaggttctagca

U2 Fw, ccttttggctaagatcaagtg; U2 Rv, agcaagctcctattccaactc

Actb Fw, agccttccttcttgggtatgg; Actb Rv, tggtaccaccagacagcactgt

Gapdh Fw, cctcgtcccgtagacaaaatg; Gapdh Rv, tctccactttgccactgcaa

**human XIST**

forward primer; reverse primer

1Fw, tgattcccttcccctctgaac; 1Rv, aaaaagcaggtatccgaagcc

2Fw, aaggtgggatggacagtgct; 2Rv, tgccatgctaattcacccag

3Fw, cccagcttctctcgaaagtca; 3Rv, agatcagcaatgccaagggta

4Fw, gcagcgctgactacctgag; 4Rv, gtcccactgctgttatgcaa

5Fw, caaggtccctgcatcatctt; 5Rv, ccaagtaccccctgctgtaa

6Fw, cttggcaaagcagcaggagt; 6Rv, gagcaaggaagcgggattct

7Fw, cgtaatcgcacactgctcat; 7Rv, atgtggagaggaccctcctt

8Fw, gcaggggtactgaatcaccaag; 8Rv, agggaaaggaagattggaggtg

9Fw, tgcgccagtgtgtaaaatcac; 9Rv, aagggaagagggagacagcac

10Fw, tcctacaagcagtgcagagagc; 10Rv, tcctcaagtgctagagtgccag

11Fw, ttgcatatgtgggcaagtgt; 11Rv, ctccacaatgcttgctctga

12Fw, cagcaacagcttccttctttga; 12Rv, actatgagcagggagttcaggc

13Fw, ccttgacctggcctacagaa; 13Rv, cctcaggacccagaatggta

14Fw, ggagggagcaaggttgaaga; 14Rv, catccccagctgaagaaagg

**FLAG-HA hnRNP U knockin by CRISPR/Cas**

hnRNPU-CRI-F, caccgcaggcgaagaactcatgttg

hnRNPU-CRI-R, aaaccaacatgagttcttcgcctgc

sgRNA-(F+E)-F1, gtttaagagctatgctggaaacagcatagcaagtttaaataaggctagtccgttatcaacttgaa

sgRNA-(F+E)-F2, aaagtggcaccgagtcggtgctttttttctcgagtactaggatccattaggcggccgcgtggataaccgtattaccgcctctagaggtac

sgRNA-(F+E)-R1, ctttttcaagttgataacggactagccttatttaaacttgctatgctgtttccagcatagctctt

sgRNA-(F+E)-R2, ctctagaggcggtaatacggttatccacgcggccgcctaatggatcctagtactcgagaaaaaaagcaccgactcggtgcca

* The mutation sites are underlined.

hnRNPU-FLAGHA-KI, cctccttcagctccgacaccttcagcttcttgacattaacaggcgaagaactcatggctgaaccgcctccaccAGCGTAATCTGGAACATCGTATGGGTAtcctccagcggcgatCTTGTCGTCATCGTCTTTGTAGTCcatgttgagggccccggttccccgctgactgctggctccctcggcctgtggcggcggcg

**The homology arms are underlined. FLAG and HA tags are shown as capital letters.

hnRNPU-F, gtctcctcagccacctgttg

hnRNPU-R, ttgccttttgacacaccgta
